# Ligase 3 prevents oxidative strand break-induced mitochondrial DNA loss but is not essential for replicative circularization

**DOI:** 10.1093/nar/gkaf1000

**Published:** 2025-10-14

**Authors:** Genevieve Trombly, Afaf Milad Said, Alexei P Kudin, Kerstin Hallmann, Anano Kakabadze, Viktoriya Peeva, Kerstin Becker, Karl Köhrer, Gábor Zsurka, Wolfram S Kunz

**Affiliations:** Institute of Experimental Epileptology and Cognition Research, Medical Faculty, University of Bonn, Bonn 53127, Germany; Institute of Experimental Epileptology and Cognition Research, Medical Faculty, University of Bonn, Bonn 53127, Germany; Institute of Experimental Epileptology and Cognition Research, Medical Faculty, University of Bonn, Bonn 53127, Germany; Institute of Experimental Epileptology and Cognition Research, Medical Faculty, University of Bonn, Bonn 53127, Germany; Institute of Experimental Epileptology and Cognition Research, Medical Faculty, University of Bonn, Bonn 53127, Germany; Institute of Experimental Epileptology and Cognition Research, Medical Faculty, University of Bonn, Bonn 53127, Germany; Cologne Center for Genomics, Medical Faculty, University of Cologne, Cologne 50931, Germany; Biological and Medical Research Centre (BMFZ), Genomics & Transcriptomics Laboratory, Heinrich Heine University Düsseldorf, Düsseldorf40225, Germany; Biological and Medical Research Centre (BMFZ), Genomics & Transcriptomics Laboratory, Heinrich Heine University Düsseldorf, Düsseldorf40225, Germany; Institute of Experimental Epileptology and Cognition Research, Medical Faculty, University of Bonn, Bonn 53127, Germany; Department of Epileptology, University Hospital Bonn, Bonn 53127, Germany; Institute of Experimental Epileptology and Cognition Research, Medical Faculty, University of Bonn, Bonn 53127, Germany; Department of Epileptology, University Hospital Bonn, Bonn 53127, Germany

## Abstract

The mitochondrial isoform of LIG3 is proposed to catalyze both circularization of newly replicated mitochondrial DNA (mtDNA) and rejoining of free mtDNA strands in base excision and single-strand break repair. Inactivation of LIG3 has been reported to cause embryonic lethality in mice due to loss of mtDNA. Here, we applied genome editing to inactivate LIG3 in HEK 293 cells and observed only a moderate decrease of mtDNA copy numbers. BrdU incorporation experiments confirmed ongoing synthesis of intact supercoiled mtDNA. Using ultra-deep long-read sequencing of isolated mtDNA, we detected increased frequencies of single-strand and double-strand breaks clustering at sites with high GC-content, as well as hallmarks of accelerated degradation of linear mtDNA. This is likely due to the missing repair of intrinsic oxidative single-strand breaks, since the frequency of detected single-strand breaks was dependent on oxygen tension and on expression levels of enzymes involved in ROS (reactive oxygen species) defense. Exogenous oxidative challenge, that resulted in transient mtDNA damage in wild-type cells, caused dramatic mtDNA loss in *LIG3^−/−^* cell lines. Thus, our data provide evidence for the pivotal role of LIG3 in preventing mtDNA loss after oxidative damage and corroborate the hypothesis that oxidative strand break-induced mtDNA degradation is highly relevant for mtDNA turnover *in vivo*.

## Graphical Abstract

**Graphical Abstract:**
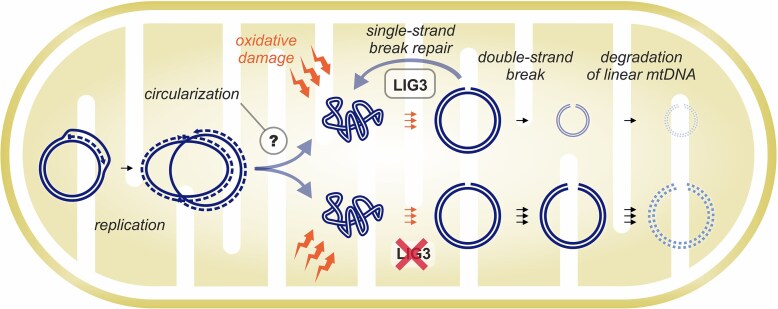

## Introduction

Maintenance of the mitochondrial DNA (mtDNA) bears fundamental differences to safeguarding of the nuclear genome. These are consequences of two specific features of the mtDNA. First, mtDNA in bilaterian animals is circular (in contrast to linear nuclear chromosomes), thus, its synthesis requires joining of ends of newly replicated full-length mtDNA strands. Second, most cells contain hundreds to thousands copies of the mtDNA, which means that damage of a single copy has only limited influence on cellular function. On nuclear chromosomes, extensive redundant repair pathways ensure that none of the two available copies is rendered dysfunctional after damage. In case of the multicopy mitochondrial genome, it might be more secure and cost-efficient to degrade the damaged DNA molecule and recycle its nucleotides for replication from intact templates rather than to initiate an error-prone reconstruction of the original molecule [1]. We and others showed that components of the replication machinery are involved in the removal of linear mtDNA, i.e. DNA molecules that carry double-strand breaks (DSBs) [2–4].

Single-strand breaks (SSBs), on the other hand, are much more frequent mtDNA lesions and can be repaired. It has been shown that all enzymatic activities required for the base excision repair (BER) pathway are present in mitochondria [5]. One of these is the mitochondrial isoform of LIG3—the only mtDNA ligase—which is proposed to perform both the rejoining of free mtDNA strands in the final step of the BER pathway and the circularization of newly replicated mtDNA. It has been demonstrated that LIG3 is essential for the viability of mammals, since early embryonic lethality was observed after targeted inactivation in mice [6]. This phenomenon has been proposed to be related to the mitochondrial isoform of LIG3, since viability could be rescued by targeting various kinds of ligase activities to mitochondria, like either LIG3 itself [7, 8] or the mammalian ligase 1, *Chlorella* virus DNA ligase, or the NAD^+^-dependent LigA of *Escherichia coli* [9, 10]. Genetic inactivation of the nuclear isoform of *Lig3* in mice has been observed to have only minor impact on mouse physiology [11].

Lack of LIG3 in cultured mouse embryonic fibroblasts leads to almost complete loss of mtDNA, but these cells (like other mtDNA-depleted cells) have been shown to be viable in a growth medium that is supplemented with uridine and pyruvate [12]. The mtDNA loss was attributed to the role of LIG3 in mtDNA replication: the end ligation reaction required for completing the synthesis of the circular genome [12, 13]. However, an important role of LIG3 for protection against oxidative stress due to its role in mitochondrial BER pathway has been also noted [14]. In humans, biallelic loss-of-function mutations have been found to cause neurogastrointestinal encephalomyopathy [15] or a myopathic mtDNA depletion syndrome [16]. However, only combinations of truncating mutations on one allele with amino acid changes on the other allele have been described in these patients, suggesting only partial loss of LIG3 function in these disease conditions, which potentially explains the difference to the embryonal lethality in mice.

In this study, we demonstrate that LIG3 inactivation in human HEK 293 cells does not prevent generation of supercoiled circular mtDNA and has only modest effects on baseline mtDNA replication. This is in contradiction with the suggested crucial role of LIG3 in post-replicative sealing of nascent DNA strands and points to the existence of alternative enzymatic activities. On the other hand, we confirm the critical role of LIG3 in rescuing mtDNA after oxidative damage and, in general, in ROS (reactive oxygen species)-dependent mtDNA turnover.

## Materials and methods

### Cell growth conditions

HEK 293 cells were commercially obtained from ATCC (Nr. CRL-1573). They were cultured in Dulbecco’s modified Eagle’s medium with high glucose (4.5 g/l), 1 mM sodium pyruvate, sodium bicarbonate, and GlutaMAX™ (Gibco) or stable Glutamine (PAN Biotech). This medium was supplemented with 10% heat-inactivated tetracycline-free FBS (PAN Biotech), 100 U/ml penicillin and 100 μg/ml streptomycin (Gibco), and 50 μg/ml uridine (Sigma–Aldrich). Alternatively, cells were grown in the same medium without pyruvate and uridine. Cells were maintained in an HERAcell 150 (Thermo Fisher Scientific) incubator at 37°C in humidified atmosphere with 10% CO_2_ and 19% O_2_. When indicated, the atmosphere in the incubator was supplied with extra N_2_ to reduce the O_2_ content to 2% and cells were cultured for 14 days under these conditions. Transient oxidative stress was induced on cells at 90% confluence by applying 1 mM H_2_O_2_ (Honeywell) to the growth medium. We have demonstrated previously that H_2_O_2_ vanishes within minutes in medium containing pyruvate and growing cells [17]. Viability was determined by adding 0.1% erythrosin B (Sigma–Aldrich) to cells suspended in 1× phosphate buffered saline (PBS; Gibco) and counting stained and non-stained cells in a Neubauer hemocytometer (Marienfeld).

### Genome editing by CRISPR–Cas9

gRNA and sequencing primer sequences were gathered from published data from Horizon Discovery Ltd of their successful HAP1 knockout cell line in the third exon of the *LIG3* gene (Supplementary Table S1). The target sequence was extended to generate overhangs that anneal into the linearized CRISPR Nuclease Vector (GeneArt). Oligonucleotides were purchased from Biomers.net. Transfection and single cell dilution were performed essentially as described in [3]. Polymerase chain reaction (PCR) followed by Sanger sequencing was used to determine whether desired mutations are present in *LIG3*.

### Western blotting

Cells were sonicated (2 × 15 s at 8% amplitude) in cell lysis buffer containing 20 mM Tris–HCl (pH 7.4), 150 mM NaCl, 1mM ethylenediaminetetraacetic acid, 1mM EGTA, 1% Triton, and protease inhibitor cocktail (Roche). Protein amount was determined using the bicinchoninic acid (BCA) assay. Protein extracts (15–40 μg) in Laemmli buffer were resolved on a 10% polyacrylamide gel and transferred to a Hybond™ P 0.45 PVDF-membrane (Amersham). Membranes were incubated overnight at 4°C with the primary antibodies: β-actin (GeneTex, 1:10 000) and LIG3 (Sigma–Aldrich, 1:500). Detection was performed with horseradish peroxidase-conjugated secondary antibodies (goat anti-rabbit IgG–Peroxidase, Sigma–Aldrich, 1:20 000) and SuperSignal West Pico Plus chemiluminescent substrate (Thermo Scientific) on a ChemiDoc Imaging System (Bio-Rad).

### Mitochondrial enzyme activities

We measured activity of the mitochondrial enzymes cytochrome *c* oxidase (COX) and citrate synthase using standard methods [18]. All measurements were performed at 30°C with a dual wavelength spectrophotometer (Aminco DW-2000, SLM Instruments, Rochester, NY, USA). Shortly, the cytochrome *c* oxidase activity was measured in 0.1 M potassium phosphate buffer (pH 7.4), 0.02% laurylmaltoside, and 200 μM reduced cytochrome *c*. The reaction was started with sample addition and the oxidation of ferrocytochrome *c* was monitored at the wavelength pair 510/535 nm (ϵ_red–ox_ = 5.9 mM^−1^ cm^−1^).

### Isolation of mitochondria

Mitochondria were isolated from whole cells via differential centrifugation based on the protocol outlined in [19]. Briefly, ice-cold isolation buffer (IB; 210 mM mannitol, 70 mM sucrose, and 5 mM HEPES–KOH, pH 7.2) was bubbled with argon gas (Linde) for a minimum of 30 min to remove any oxygen in the solution that would result in additional oxidative damage and supplemented with 0.25% bovine serum albumin (BSA; PAN Biotech). All steps were performed at 4°C. Trypsinized cells (3–9 × 10^8^) were collected by centrifugation at 500 × *g* for 10 min and washed once in 1× PBS. Cell pellet was resuspended in IB + BSA (4 ml for each gram) and water-soluble digitonin (SERVA) was added at 0.1 mg/ml final concentration. Successful cell permeabilization was confirmed by 0.1% erythrosin B staining. If necessary, additional digitonin was applied in 0.05 mg/ml increments. Cell suspension was diluted with IB + BSA to double the volume and centrifuged at 3000 × *g* for 5 min. Pellet was resuspended in IB + BSA (5 ml for each original gram of cells) and homogenized with a Dounce-homogenizer applying two to five strokes. The volume of the homogenate was tripled and it was centrifuged twice at 1000 × *g* for 5 min to remove nuclei. The supernatant was centrifuged at 10 000 × *g* for 30 min, the brown mitochondrial pellet was washed three times in IB + BSA, and finally dissolved in 900 μl IB without BSA. To remove adherent proteins and nuclear DNA, 15 μl proteinase K (Qiagen, >600 mAU/ml) was added and the mitochondrial suspension was incubated at 26°C for 1 h. The mitochondrial pellet was collected by centrifugation at 10 000 rpm for 15 min in a microcentrifuge and washed twice in IB + BSA.

### DNA isolation

DNA from harvested cells or mitochondria-enriched fractions was isolated using the QIAamp DNA Mini Kit (Qiagen) according to the manufacturer’s protocol for tissue isolation. DNA concentration was measured using a Qubit™ 4 fluorometer (Invitrogen). Qubit™ assay tubes and Qubit™ 1× dsDNA HS Assay Kit (Invitrogen) were used according to the manufacturer’s protocol.

### Southern blotting

One micrograms of total DNA was digested with MluI restriction endonuclease (Fermentas). Digested DNA was separated on a 0.6% agarose gel containing 0.5 μg/ml ethidium bromide at 40 V overnight along with DIG-labeled DNA Molecular Weight Marker II (Roche). Gels were alkaline treated and neutralized. DNA was blotted to Zeta-Probe Membranes (Bio-Rad) and immobilized by baking at 80°C for 30 min. Blots were hybridized overnight at 48°C with PCR-generated digoxigenin-labeled probes (PCR DIG Probe Synthesis Kit, Roche) for the mitochondrial genome (using primers MT-ND5_F and MT-ND5_R; Supplementary Table S1) or for nuclear 18S ribosomal RNA (rRNA) gene (using primers RNA18S_F and RNA18S_R) probes. Chemiluminescent detection with anti-DIG-AP antibody F_ab_ fragment (Roche) and CSPD (Roche) was performed and signal was recorded on a ChemiDoc Imaging System (Bio-Rad). Prior to relabeling, blots were stripped with 0.2 M NaOH + 0.1% sodium dodecyl sulfate at 37°C.

### Quantitative PCR

Quantitative PCR was used to determine the mtDNA copy numbers as described previously [3, 17]. Primers MT-ND1_F and MT-ND1_R (Supplementary Table S1) were used to amplify a segment of the minor arc region of the mtDNA. Amplifications were performed on a CFX96 real-time PCR system (Bio-Rad, Munich, Germany) using 2× SYBR Green qPCR Master Mix (Bimake) or Luna Universal qPCR Master Mix (New England Biolabs, Frankfurt am Main, Germany) under the following conditions: 95°C for 15 min, and 45 cycles of 95°C for 15 s and at a primer-specific annealing temperature for 1 min. Ct values were defined at the inflection points of fitted four-parameter Chapman curves and were compared with those of the single-copy nuclear gene *KCNJ10* amplified by primers KCNJ10_F and KCNJ10_R (Supplementary Table S1). For each sample and each primer pair, triplicate reactions were performed at two different concentrations of template DNA.

### Transcript analysis

Total RNA of pelleted cells was isolated with the RNeasy Mini Kit (QIAgen, Hilden, Germany). Random primed complementary DNA (cDNA) was produced by transcription of 1 μg RNA of each sample using the iScript Select cDNA Synthesis Kit (Bio-Rad Laboratories, Munich, Germany). Quantitative PCR reactions were performed on a CFX96 real-time PCR system (Bio-Rad, Munich, Germany) using SYBR Green qPCR Master Mix (Bimake, Munich, Germany) and using following thermal cycling conditions: *NFE2L2*, *POLG*, *MGME1*, *TOP3A*, *LIG3*, *GAPDH*, 95°C for 15 min, and 45 cycles of 95°C for 15 s and 62.5°C for 45 s; *GSR*, *TXN*, 95°C for 15 min, and 45 cycles of 95°C for 15 s and 62°C for 30 s; *PRDX1*, 95°C for 15 min, and 45 cycles of 95°C for 15 s and 61°C for 30 s. Ct values were defined at the inflection points of fitted sigmoid curves (four-parameter Chapman curves) and were compared with those of the reference gene *GAPDH*. For each sample and each primer pair, triplicate reactions were performed at two different concentrations of template cDNA. Primer sequences are provided in Supplementary Table S1.

### BrdU incorporation and decay experiments

Incorporation of 5-bromo-2′-deoxyuridine (BrdU) into mtDNA can be used to visualize replication and repair kinetics [20]. One hour before applying BrdU to cells, aphidicolin (Sigma–Aldrich) dissolved in dimethylsulfoxide (DMSO) was added to the cell culture medium at a final concentration of 20 μM in order to allow for the halting of nuclear DNA replication [21]. Water-dissolved BrdU (Roche) was then added to the cell culture medium to a final concentration of 10 μM after which cells were harvested at indicated time points. BrdU-labeled mtDNA was visualized by southwestern blotting and chemiluminescent detection using an anti-BrdU primary antibody (mouse IgG; Sigma–Aldrich) and a goat anti-mouse HRP conjugate (Bio-Rad) as secondary antibody. The chemiluminescent signal was developed using the Clarity Western ECL Substrate Kit (Bio-Rad) and the signal was recorded on a ChemiDoc Imaging System (Bio-Rad). In order to investigate the decay of labeled mtDNA, cells were first allowed to incorporate BrdU for 24 h in the presence of aphidicolin, growth medium was then replaced with a medium containing neither BrdU nor aphidicolin, and cells were harvested at indicated time points.

### mtDNA depletion and repopulation experiments

Depletion and repopulation of mtDNA in the different HEK 293 clones were performed by treatment with 2′,3′-dideoxycytidine (ddC) essentially as described in [22]. This nucleoside is imported into the mitochondrial compartment, phosphorylated, and incorporated into the mtDNA [23], where it causes strong replication inhibition by preventing elongation of nascent mtDNA. The cells were treated for 12 days with the ddC concentration indicated to deplete their mtDNA; thereafter, ddC was omitted from the incubation medium to allow repopulation. Cells were collected in 2-day intervals by trypsinization and centrifugation at 500 × *g* for 10 min. DNA isolation and mtDNA copy number determination by qPCR were performed as described above.

### Long-read single-molecule (PacBio) sequencing of isolated mtDNA

For long-read single-molecule sequencing, 1.5 μg of isolated mtDNA was linearized by the single-cutter restriction endonuclease EagI (New England Biolabs). Samples were RNase digested and purified on 0.8X AMPure PB beads prior to library preparation with the Express Template 2.0 Kit (Pacific Biosciences) according to the protocol “Procedure & Checklist—Preparing Multiplexed Microbial Libraries Using SMRTbell Express Template Prep Kit 2.0” (Version 04, November 2019) without additional DNA shearing, starting with removal of single-strand overhangs, and using the Barcoded Overhang Adapter Kit 8A or B (Pacific Biosciences) for adapter ligation. After library preparation, six libraries were pooled equimolarly and the pools size selected with diluted AMPure PB beads to remove fragments <3 kb. Library pools were quantified (Qubit) and analyzed for final fragment size distribution (Fragment Analyzer). Sequencing primers and polymerase were successively annealed and bound to the libraries and each pool was sequenced on one 8M SMRT cell on a Sequel II Instrument (Pacific Biosciences) with 30 h movie time and 2 h pre-extension. Circular consensus sequence reads were generated and demultiplexed with SMRT Link v9 (Pacific Biosciences). To aid in detection of SSBs, 350 ng mtDNA was treated with 100 U S1 Nuclease (Thermo Scientific) for 15 min at 37°C prior to linearization. 7–29 × 10^4^ reads were obtained per sample of which 76%–92% aligned to the human mitochondrial genome. Alignment of long reads was performed using an in-house R script based on the *pairwiseAlignment* function of the *Biostrings* package (version 2.64.1) with parameters *gapOpening* = 5, *gapExtension* = 2 and nucleotide substitution matrix of *match*= 1, *mismatch* = −3, *baseOnly* = true. In order to account for varying experimental read-length bias, mitochondrial and nuclear fragment counts were normalized to the nuclear fragment size distribution of an experiment with the highest proportion of large fragments. End frequencies were normalized to average sequencing depth.

### Statistical analyses

Two-tailed Student’s *t*-test or one-way ANOVA with Bonferroni post hoc test was performed as indicated.

## Results

### LIG3 is essential for recovery of mtDNA after oxidative damage but dispensable for *de novo* synthesis of circular mtDNA

**Figure 1.**
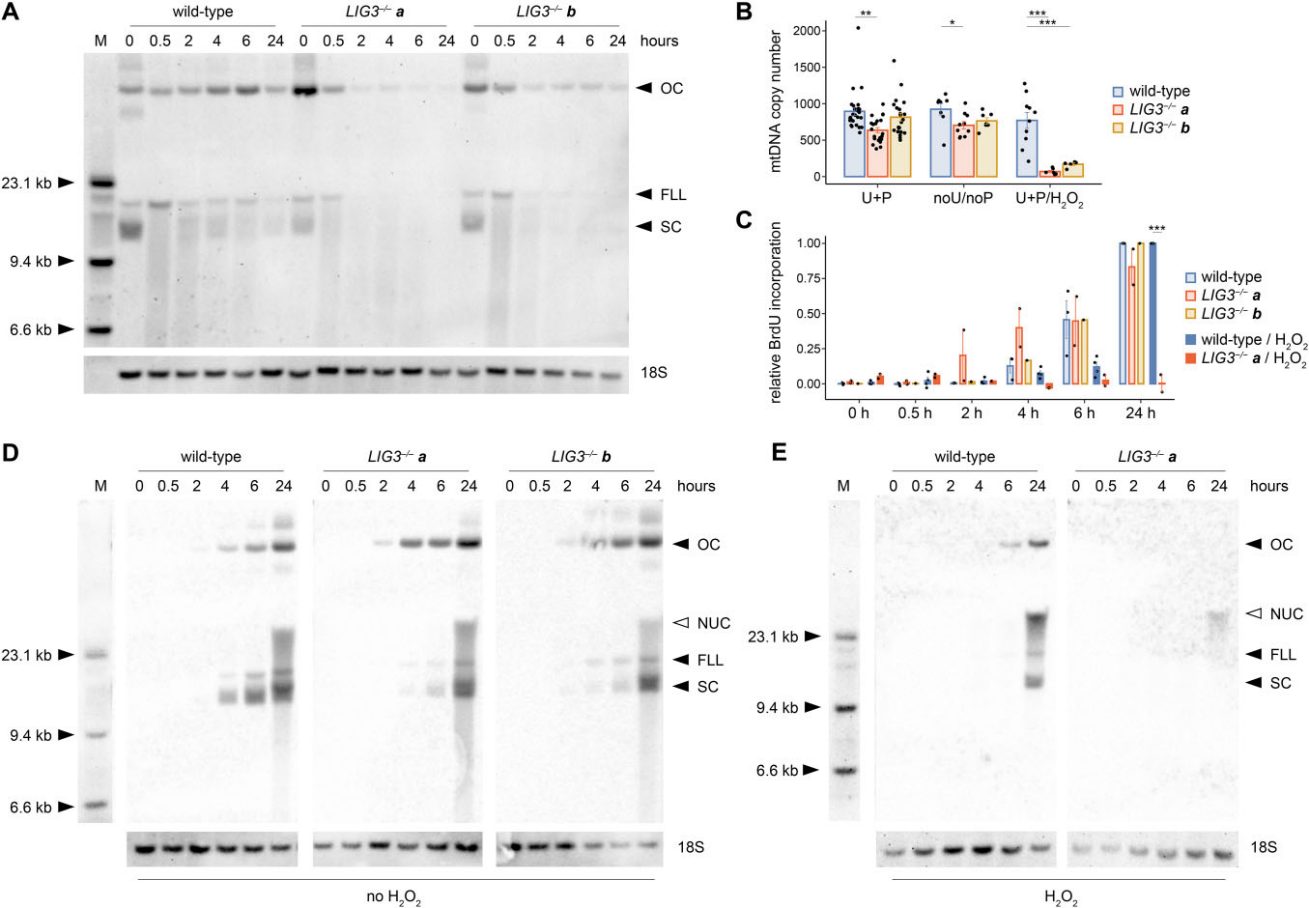
Hydrogen peroxide exposure induces mtDNA loss in *LIG3^−/−^* cells. (**A**) Southern blot of MluI-digested DNA harvested from wild-type and *LIG3^−/−^* cells at different time points after application of 1 mM H_2_O_2_. mtDNA was labeled with a probe located in the *MT-ND5* gene (upper panel), then blots were relabeled with a probe specific for nuclear 18S rRNA genes as loading control (lower stripe). The MluI restriction endonuclease cuts within nuclear 18S rRNA genes, but has no recognition site in the human mitochondrial genome. Three main conformations of native mtDNA are indicated by arrowheads: OC, open circle; FLL, full-length linear; SC, supercoiled circular genome. The identity of the various mtDNA conformations was confirmed by treating native mtDNA with Nb.BbvCI nickase (Supplementary Fig. S3). The smear in samples obtained after H_2_O_2_ treatment represents linear mtDNA fragments of various sizes. (**B**) Copy numbers of mtDNA in wild-type and *LIG3^−/−^* cells as determined by quantitative PCR. U+P, cells grown in medium supported by uridine and pyruvate. noU/noP, cells grown at least 1 week in medium without uridine and pyruvate. U+P/H_2_O_2_, cells grown in medium supported by uridine and pyruvate 24 h after exposure to 1 mM H_2_O_2_. (**C**) Quantification of relative intensities of mitochondrial BrdU incorporation at different time points with or without H_2_O_2_ exposure. Values were normalized to the 24-h intensity of the corresponding wild-type sample. Representative southwestern blots are shown in panel (**D**) for BrdU incorporation without H_2_O_2_ treatment and in panel (**E**) after an H_2_O_2_ pulse. Despite the presence of nuclear DNA synthesis inhibitor aphidicolin, a residual nuclear BrdU incorporation is detectable and indicated by an empty arrowhead at the top of a smeared signal (NUC). 0, untreated cells. M, molecular weight marker. Error bars, SEM. *P < .05, **P < .01, ***P < .001 by one-way ANOVA and Bonferroni post hoc test.

In order to investigate the role of the ligase LIG3 in the maintenance of human mtDNA, we used CRISPR–Cas9 genome editing to genetically inactivate the *LIG3* gene in HEK 293 cells (Supplementary Fig. S1A and B). We confirmed the lack of the LIG3 protein in two independent clones by western blotting (Supplementary Fig. S1C). Since previous studies reported severe depletion of mtDNA in *Lig3^−/−^* mouse embryonic fibroblasts [12], we generated and maintained the *LIG3^−/−^* HEK 293 cells in a medium containing high glucose, pyruvate, and uridine, which enables cell growth even in case of complete lack of mtDNA [12]. In opposite to previous reports in mouse fibroblasts [12], we did not detect significant decrease in cytochrome *c* oxidase activity in *LIG3^−/−^* cells in comparison to wild-type cells (Supplementary Fig. S2; 0 h). We observed only a moderate decrease in mtDNA copy number (Fig. 1B) in one HEK 293 cell clone (635 ± 171, clone *a*; control 895 ± 277; *P* = .0017; one-way ANOVA and Bonferroni post hoc test) and no significant alteration in the other clone (815 ± 260, clone *b*) in supportive medium. Copy numbers of mtDNA were not affected when omitting uridine and pyruvate from the growth medium (Fig. 1B). Therefore, we concluded that the synthesis of circular mtDNA is not strictly dependent on LIG3 function in HEK 293 cells under normal growth conditions.

Next, we exposed cells to a short hydrogen peroxide pulse to introduce transient oxidative damage without affecting cell survival (Supplementary Fig. S4). We visualized the different conformations of mtDNA by non-denaturing agarose gel electrophoresis and Southern blotting in the presence of ethidium bromide (Fig. 1A). In accordance with our previous results [17, 24], wild-type cells showed an instant decline of the intact supercoiled circular mtDNA 30 min after H_2_O_2_ exposure, which was partially reversed within 2 h. At the same time, we also observed transiently increased intensities of the band representing full-length linear mtDNA as well as the smear representing fragmented linear mtDNA. These alterations were reversed after a 24-h recovery time in wild-type cells. In *LIG3^−/−^* cells, no fast recovery of supercoiled mtDNA was observed after H_2_O_2_-induced oxidative damage. Instead, a dramatic loss of all mtDNA species occurred within 24 h (Fig. 1A and B). These data confirm the pivotal role of LIG3 in recovering mtDNA after acute oxidative damage, as reported previously [25, 26].

We aimed to compare the dynamics of *de novo* mtDNA synthesis in *LIG3^−/−^* and wild-type cells by measuring BrdU incorporation [21]. To this end, we incubated cells with aphidicolin for 1 h to halt nuclear DNA replication and then with aphidicolin + BrdU for an additional 24 h to label newly synthesized mtDNA. We did not observe an altered net BrdU incorporation rate in *LIG3^−/−^* cells in comparison to wild-type cells (Fig. 1C and D). However, when investigating the individual species of native undigested mtDNA, we noted an increased incorporation in relaxed open circle mtDNA species at the expense of supercoiled species in *LIG3^−/−^* cells in comparison to wild-type cells (Fig. 1D). This is in agreement with our Southern blotting results, where we detected elevated steady-state levels of open circle mtDNA in *LIG3^−/−^* cells before exogenous oxidative damage (Fig. 1A, 0 h, and Supplementary Fig. S5). When applying BrdU in combination with H_2_O_2_, no BrdU incorporation in mtDNA was observed within the first 24 h in *LIG3^−/−^* cells, in opposite to wild-type cells (Fig. 1C and E). This indicates a blockade of mtDNA replication after oxidative damage in *LIG3^−/−^* cells likely due to lack of intact template molecules.

### mtDNA in *LIG3^−/−^* cells contains clusters of single-strand breaks

**Figure 2.**
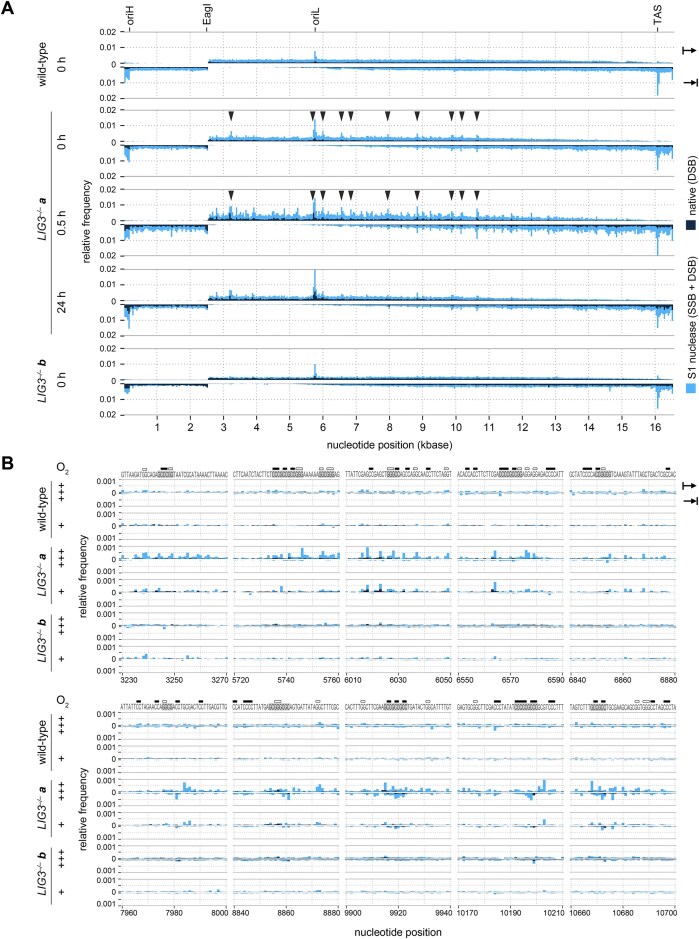
Increased frequency of mtDNA strand breaks in *LIG3^−/−^* clone *a* cells as detected by long-read deep sequencing. (**A**) Overview of all detected mtDNA fragment ends. Values are shown using 40-nucleotide binning. Upper panels show upstream ends (according to numbering of the human mitochondrial genome) and lower panels show downstream ends, as indicated on the top right side. Artificially introduced ends at the immediate vicinity of the cutting site of the single-cutter EagI restriction endonuclease (positions 2550–2585) are excluded. Black; ends detected in native DNA (representing DSBs). Blue; ends detected in S1 nuclease-treated DNA (representing a sum of DSBs and SSBs). Note the lack of signal in the ∼3-kb vicinity of the EagI cutting site, which is due to size exclusion during library preparation. Arrowheads; prominent S1 nuclease-generated ends in *LIG3^−/−^* clone *a* cells under baseline conditions. Displayed ends were selected from the top 15 bins with the highest background-subtracted residuals at upstream ends, excluding the regions D-loop (positions 16030–570) and oriL (positions 5770–5820). Detailed views of corresponding regions are shown in Supplementary Fig. S5. Note that these ends are present in *LIG3^−/−^* clone *a* cells even before H_2_O_2_ treatment (0 h) and further increased after H_2_O_2_ exposure (0.5 h), while they are not prominent in wild-type and *LIG3^−/−^* clone *b* cells. Ends in *LIG3^−/−^* clone *a* cells 24 h after H_2_O_2_ treatment are shown for completion (24 h), however, the majority of mtDNA is already purged at this time point. oriH, oriL; replication origins of the mitochondrial genome. TAS; termination-associated sequence. (**B**) Detailed view of mtDNA regions that show increased frequency of mtDNA SSBs in *LIG3^−/−^* clone *a* cells at two different oxygen tension conditions. Gray shading highlights GC-stretches. Filled and empty boxes above the sequences indicate sequence motifs that can potentially be parts of G-quadruplex structures on the heavy and the light strand, respectively. +++, samples from cells cultured in a 19% oxygen containing atmosphere; +, samples from cells cultured in a 2% oxygen containing atmosphere.

The increased frequency of relaxed open circle mtDNA species in *LIG3^−/−^* cells, as observed in Southern blotting (Fig. 1A) and BrdU incorporation experiments (Fig. 1D), likely points to an accumulation of SSBs of the mtDNA in the absence of LIG3. To confirm this, we performed ultra-deep long-read sequencing of restriction endonuclease-linearized isolated mtDNA, as we described previously [17]. In order to detect both SSBs and DSBs, we investigated samples in parallel with and without S1 nuclease treatment. S1 nuclease converts SSBs (nicks and gaps) into DSBs [27], thus the mtDNA ends in S1 nuclease-treated DNA samples represent a sum of SSBs and DSBs [17]. Long-read sequencing of mtDNA from wild-type HEK 293 cells showed prominent SSBs at the replication origins oriL and oriH, as well as in the vicinity of the termination-associated sequences (TASs), and a low level of minor SSBs distributed all along the mitochondrial genome. This finding is consistent with the presence of replication intermediates, as predicted by the classical asynchronous strand-displacement mechanism [28]. A similar pattern and frequency of SSBs was present in *LIG3^−/−^* clone *b* cells. In *LIG3^−/−^* clone *a* cells, however, we detected an increased frequency of SSBs at specific sites (Fig. 2A, arrowheads, and Supplementary Fig. S5). In addition to prominent sites at the oriL, oriH, and TAS regions mentioned above, SSBs clustered at sites displaying long GC-stretches and having the potential to form G-quadruplex structures [29] (Fig. 2B and Supplementary Fig. S5). Application of a hydrogen peroxide pulse increased the frequency of both SSBs and DSBs, which is in concert with our previous results in wild-type cells [17]. Interestingly, the pattern of SSB clusters visible in *LIG3^−/−^* clone *a* cells observed after 30 min H_2_O_2_ treatment (Supplementary Fig. S6) was very similar to the pattern under baseline conditions and was also localized to GC-rich stretches of mtDNA (Fig. 2A, stars). This raises the hypothesis that intrinsic oxidative stress might be responsible for the generation of observed baseline SSB clusters in *LIG3^−/−^* clone *a* cells. This point of view is further corroborated by findings that a reduction of the oxygen content of the cell growth atmosphere from 19% to 2% resulted in reduced frequencies of clustered SSBs in both *LIG3^−/−^* clones (Fig. 2B). Whether the observed SSBs had been generated by direct oxidative attack of the sugar-phosphate backbone or by enzymatic removal of oxidized bases and abasic sites cannot be assessed by our experimental setting and requires further investigation.

Although the limited number of repeats prevents us from making quantitative statements about the prominent ends in the oriH, TAS, and oriL regions, we observed that all *LIG3^−/−^* samples showed higher peaks at oriL than the corresponding wild-type cells (Fig. 2A). We did not observe similar tendencies in the oriH or TAS regions.

### *LIG3^−/−^* cells show elevated mtDNA turnover

**Figure 3.**
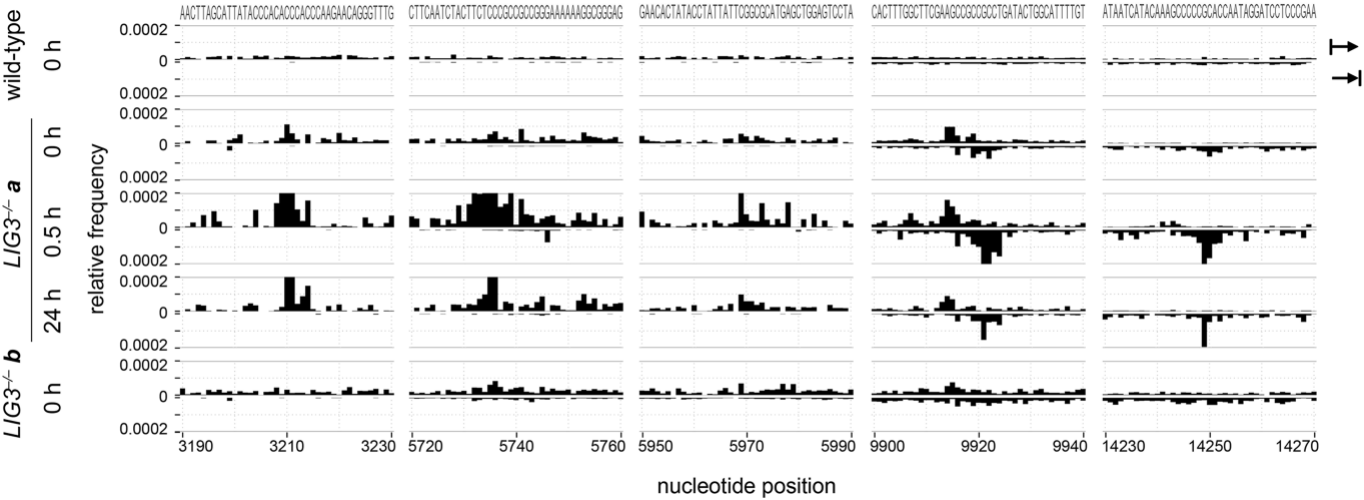
Double-stranded mtDNA ends at prominent degradation stalling sites. Positions were identified previously by introducing massive uniform mtDNA DSBs using a mitochondria-targeted restriction endonuclease (fig. 3D and E in [3]). Ends of this type are present in *LIG3^−/−^* clone *a* cells not only after H_2_O_2_ treatment (0.5 h, 24 h) but also at baseline condition (0 h). Note that identical data ranges are shown to facilitate comparison, which results in truncation of large peaks in the 0.5-h and 24-h *LIG3^–/–^* clone *a* samples. The detected maxima at specific sites are 3210 upstream, 0.00162 (0.5 h) and 0.00043 (24 h); 5736 upstream, 0.00112 (0.5 h) and 0.00033 (24 h); 5969 upstream, 0.00028 (0.5 h); 9921 downstream, 0.00036 (0.5 h); and 14249 downstream, 0.00048 (0.5 h).

We previously identified prominent stalling sites of mtDNA degradation by introducing uniform DSBs using a mitochondria-targeted restriction endonuclease [3] and showed later that these hallmarks of ongoing mtDNA degradation are detectable after oxidative damage in wild-type cells [17]. We now asked whether similar double-stranded mtDNA ends are also present in *LIG3^−/−^* cells. Long-read sequencing confirmed degradation-specific ends not only in H_2_O_2_-treated *LIG3^−/−^* cells but also showed an increased frequency of these ends in *LIG3^−/−^* clone *a* cells at baseline conditions (Fig. 3). This indicates that LIG3 deficient cells, that harbor elevated levels of SSBs, also show hallmarks of accelerated degradation of linear double-stranded mtDNA even without exogenous oxidative damage.

**Figure 4.**
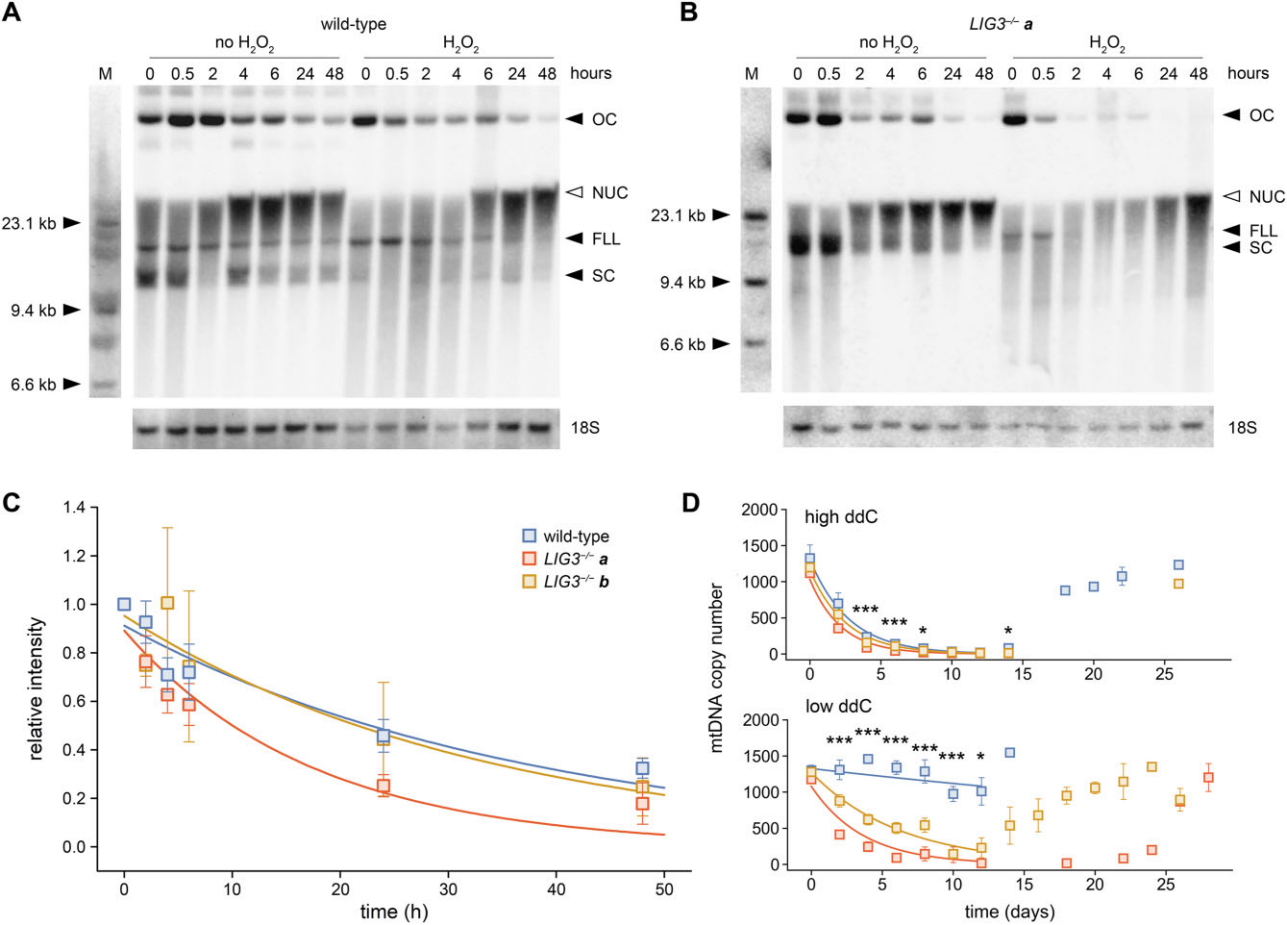
Accelerated decay of mtDNA in *LIG3^−/−^* cells detected by anti-BrdU DNA blotting and during ddC-mediated replication arrest. (**A**) Cells were first labeled for 24 h with BrdU in the presence of aphidicolin to inhibit nuclear incorporation, and then incubated in medium without BrdU and aphidicolin. DNA was digested with the MluI restriction endonuclease that cuts within nuclear 18S rRNA genes (loading control, lower stripe) but has no recognition site in the human mitochondrial genome, thus, preserves the native conformation of mtDNA. OC, open circle; FLL, full-length linear; SC, supercoiled circular genome. Note the fast reappearance of the SC signal after H_2_O_2_ treatment in wild-type cells. Residual nuclear BrdU incorporation is indicated at the top of a smeared signal (NUC, empty arrowhead). 0, untreated cells. M, molecular weight marker. (**B**) Accelerated decrease of the mitochondrial BrdU signal is detected in *LIG3^−/−^* clone *a* cells. (**C**) Quantification of the decay of mitochondrial BrdU signals in multiple experiments. Band intensities were normalized to nuclear 18S rRNA gene intensities and to the normalized value of the corresponding wild-type 0-hour sample. Blue, wild-type (*n* = 6); red, *LIG3^−/−^* clone *a* (*n* = 4); yellow, *LIG3^−/−^* clone *b* (*n* = 2). Error bars, SEM. Although an accelerated decay was observed in clone *a*, one-way ANOVA was not significant due to the inherent variability of the technique. Fitted exponential decay curves are shown. (**D**) Alterations of mtDNA copy number during ddC-induced depletion and subsequent repopulation. When applying 2 mM ddC (upper panel), all cell lines rapidly lost their mtDNA, however, *LIG3^−/−^* cells at significantly higher rates. Note that cell division rates were reduced in *LIG3^−/−^* clones during high concentration ddC treatment, precluding sampling on days 14–24. Exposure to 400 μM ddC had little effect on mtDNA copy number in wild-type cells. By contrast, *LIG3^−/−^* clones underwent pronounced mtDNA depletion, with copy number restored within 2 weeks after ddC withdrawal. Error bars, SEM. *P < .05; ***P < .001, one-way ANOVA. Fitted exponential decay curves during ddC treatment are shown. Bonferroni post hoc test indicated significant differences between the two *LIG3^−/−^* clones at both conditions of ddC treatment (low ddC: day 2, *P* = .002; day 4, *P* = .005; day 6, *P* = .003; day 8, *P* = .036; high ddC: day 4, *P* = .016).

In order to confirm ongoing mtDNA degradation at baseline conditions in *LIG3^−/−^* cells, we investigated the decay of BrdU-labeled mtDNA after removing aphidicolin and BrdU from the growth medium. We observed a slow decrease in the mtDNA signal in wild-type cells (Fig. 4A and C) resulting in approximately half of the original signal intensity after 24 h (0.46 ± 0.17). The fitted exponential decay curve is equivalent to an apparent half-life time of 26 h and roughly corresponds to dilution of labeled mtDNA by newly synthesized nonlabeled mtDNA due to cell division (on the average at ∼30 h doubling time in HEK 293 cells) (https://www.cellosaurus.org/CVCL_0045). *LIG3^−/−^* cells from clone *a* showed a more rapid decline in mtDNA signal (0.25 ± 0.09 after 24 h; half-life time of 12 h; Fig. 4B and C); however, this difference did not reach significance in the one-way ANOVA, consistent with substantial technical variability. In opposite, *LIG3^−/−^* clone *b* cells displayed a similar decay of mtDNA as wild-type cells (0.44 ± 0.33 signal intensity after 24 h; Fig. 4C). After applying H_2_O_2_, wild-type cells showed a transient fading of the supercoiled signal followed by a partial recovery within hours (Fig. 4A), while the mtDNA signal was largely lost in *LIG3^−/−^* clone *a* cells (Fig. 4B). This is in agreement with the missing BrdU incorporation after H_2_O_2_ exposure (Fig. 1C and E) and our Southern blotting results (Fig. 1A) in these cells.

We further investigated mtDNA turnover by application of ddC (Fig. 4D). This nucleoside analogue of cytosine is imported into the mitochondrial compartment, phosphorylated, and incorporated by replicative polymerase POLG into the mtDNA [23], where it causes a strong replication inhibition by preventing elongation of nascent mtDNA. During the depletion phase (in the presence of ddC), mtDNA copy number declined substantially faster in both *LIG3^−/−^* clones than in wild-type cells; moreover, clone *a* exhibited a higher depletion rate than clone *b*. Upon removal of ddC at day 12, the inhibitory block on mtDNA replication was lifted, triggering rapid replication and recovery of mtDNA copy number within 2 weeks in all three types of cells. This confirms our finding that post-replicative nick sealing remains functional without *LIG3*, while intrinsic mtDNA degradation is accelerated.

### Intracellular ROS defense modulates mtDNA turnover

**Figure 5.**
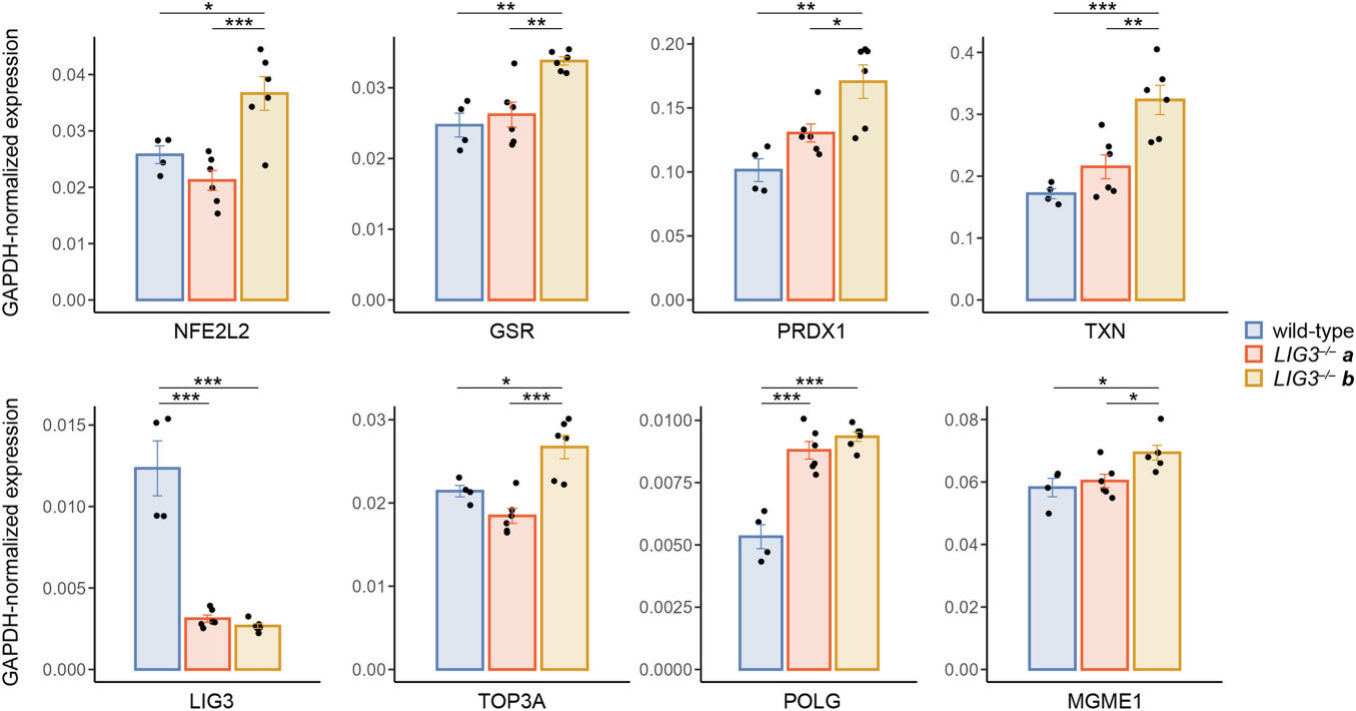
Elevated expression of genes involved in reactive oxygen species detoxification and in mtDNA maintenance in *LIG3^−/−^* clone *b* cells. Transcript abundance was determined by reverse transcription quantitative PCR and values were normalized to *GAPDH* expression. The dramatically reduced transcript level of LIG3 in both *LIG3^−/−^* clones indicates nonsense-mediated mRNA decay. Error bars, SEM. *P < .05; **P < .01; ***P < .001; one-way ANOVA with Bonferroni post hoc test.

Although the lack of the LIG3 protein was confirmed in both *LIG3^−/−^* cell lines and both *LIG3^−/−^* clones showed mtDNA loss after application of an H_2_O_2_ pulse; a number of parameters related to intrinsic mtDNA damage showed substantial differences between the two *LIG3^−/−^* clones. As described above, we did not observe SSB accumulation or steady-state copy number decrease at baseline conditions in clone *b*, in contrast to clone *a*. We hypothesized that the difference between the two *LIG3^−/−^* clones could be due to differences in resilience to baseline oxidative damage. We therefore measured transcript levels of a number of genes related to cell redox homeostasis by reverse transcription quantitative PCR. We found a 1.4–1.9-fold upregulation of the genes *NFE2L2*, *PRDX1*, *TXN*, and *GSR* in clone *b* in comparison to clone *a* or wild-type cells (Fig. 5). Importantly, NFE2L2 (Nrf2) is the major transcription factor activating intracellular ROS defense [30] and peroxiredoxin 1 (PRDX1), thioredoxin (TXN), and glutathione reductase (GSR) are important effectors under NFE2L2 control involved in cytosolic H_2_O_2_ detoxification [30]. Therefore, an elevated capacity of *LIG3^−/−^* clone *b* to detoxify intrinsic ROS is a potential reason for the attenuated level of baseline SSBs in this cell line. We detected similar increase in transcript levels of ROS defense effectors in a wild-type HEK 293 cell line that underwent single-cell cloning (Supplementary Fig. S7). This suggests that the alterations observed in *LIG3^−/−^* clone *b* reflect intrinsic heterogeneity within HEK 293 cells revealed by single-cell cloning, rather than an adaptation to LIG3 deficiency.

Additionally, we assessed the expression of selected genes involved in mtDNA replication. We detected elevated transcript levels of the mitochondrial exonuclease *MGME1* and the topoisomerase *TOP3A* in clone *b* in comparison to wild type and clone *a*. We also observed an increased expression of *POLG* in both *LIG3^−/−^* clones, which could indicate a compensatory upregulation of mtDNA synthesis under conditions of increased mtDNA turnover. However, similar elevated expression of *POLG* is also detectable in the single-cell cloned wild-type HEK 293 line (Supplementary Fig. S7). Collectively, our data indicate that the more resilient *LIG3^−/−^* clone *b* exhibits enhanced antioxidant defenses and increased mtDNA replicative capacity.

As mentioned above, our findings that HEK 293 cells can surprisingly well tolerate the loss of LIG3, at least under baseline cell growth conditions, are in contradiction with reports on mtDNA loss in *Lig3^−/−^* mouse fibroblasts [12]. We therefore asked whether cell type-specific transcriptional programs account for HEK 293 cells’ greater ability to prevent or compensate for LIG3 loss. To this end, we determined transcript levels of the above mentioned eight genes in primary human fibroblasts (Supplementary Fig. S7). We did not find significant differences between fibroblasts and wild-type HEK 293 cells for genes involved in ROS defense. On the other hand, *LIG3* itself as well as *TOP3A* and *MGME1*, all playing a role in mtDNA maintenance, showed significantly lower expression in fibroblast when compared to wild-type HEK 293 cells. This suggests that the limited capacity of fibroblasts for mtDNA replication may explain their propensity for mtDNA loss in the absence of LIG3.

### Loss of LIG3 preferentially affects mtDNA

The CRISPR–Cas9-induced frameshift mutations present in our two *LIG3^−/−^* clones are located downstream of the zinc finger domain, and thus affect both mitochondrial and nuclear isoforms of the LIG3 protein (Supplementary Fig. S1A). We therefore asked whether the observed dramatic mtDNA loss in the *LIG3^−/−^* clones after H_2_O_2_ treatment might be accompanied with similar DNA damage in the nucleus. To this end, we took advantage of the fact that the enriched mitochondrial fraction, that we used for deep sequencing, always contained traces of nuclear DNA. We found that, similar to wild-type cells [17], the H_2_O_2_ pulse leads in *LIG3^−/−^* clone *a* cells to a reduced frequency of full-length mtDNA and an increased frequency of smaller mtDNA fragments both in native DNA (indicative of DSBs) and in S1 nuclease-treated DNA (enabling to additionally detect SSBs) (Supplementary Fig. S8). No comparable alteration of the fragment size distribution was detectable in nuclear DNA fragments (Supplementary Fig. S6). The high iron content and the efficient generation of hydroxyl radical from H_2_O_2_ through Fenton reaction in mitochondria might explain the more dramatic mtDNA fragmentation in comparison to nuclear DNA [17]; and our present data demonstrate that this is also the case in cells lacking LIG3.

## Discussion

Our results provide evidence that ligase 3 is ultimately required for sealing of SSBs of mtDNA that are generated upon oxidative stress. In the lack of LIG3, this function is not compensated by other enzymatic activities in mitochondria, which, at last, results in the loss of mtDNA after oxidative damage. In opposite, the nick-sealing activity of LIG3 appears to be not the only pathway for circularization of *de novo* replicated mtDNA. *LIG3^−/−^* HEK 293 cells are still sufficient in synthesizing supercoiled mtDNA and maintaining mtDNA copy numbers that are required for normal oxidative phosphorylation. Since the mitochondrial isoform of LIG3 is the only *bona fide* mitochondrial ligase [31], circularization of newly synthesized mtDNA could alternatively be performed by the religating activity of topoisomerase TOP3A that is present in mitochondria [32, 33] and, as a type IA topoisomerase, is able to act on nicked DNA [34]. Consistent with this hypothesis, the *TOP3A* transcript is upregulated in the more resilient *LIG3*^−/−^ clone *b* (Fig. 5).

Although *LIG3^–/–^* cells are obviously proficient in generating circularized mtDNA molecules, they carry an increased proportion of open circle species (Fig. 1D and Supplementary Fig. S5). This could be either (i) due to unligated SSBs arising from intrinsic oxidative damage or (ii) because the yet uncharacterized backup mechanism for strand circularization in the absence of LIG3 may be less efficient than LIG3-mediated circularization. Importantly, due to the asymmetric nature of mtDNA replication, the two strands may undergo circularization via distinct mechanisms. It has been shown that TOP3A acts at the oriH to separate hemicatenated mtDNA circles [32, 33]. It has also been demonstrated that completion of the new L-strand is delayed and happens after the decatenation of the two daughter molecules [35]. We therefore hypothesize that H-strand circularization at oriH occurs during TOP3A-mediated decatenation, whereas sealing of the lagging L-strand is likely LIG3-dependent. This model predicts fully circularized double-stranded mtDNA when the nascent H-strand is synthesized on an intact L-strand template, but residual nicks in *de novo*-generated L-strands (i.e. in half of daughter molecules). Consistent with this, we did not observe altered SSB frequencies at oriH, while *LIG3^–/–^* cells showed a tendency toward elevated SSBs at oriL (Fig. 2A).

The finding that LIG3 is apparently dispensable for mtDNA replication in human HEK 293 cells is in contradiction with previous results in mouse embryonic fibroblast [12]. In the latter type of cells, lack of ligase 3 results in mtDNA depletion, thus, making cells deficient in oxidative phosphorylation and dependent on glycolysis. Various characteristics of specific cell types might be responsible for this difference. First, it has been suggested that alternative mechanisms of mtDNA replication, like strand-coupled replication requiring ligation of Okazaki fragments, might exist in different types of cells or in different cellular conditions [36]. This might also be valid for the last step of mtDNA replication, the circularization of *de novo* synthesized mtDNA. Whether the capability of alternative enzymatic activities (like topoisomerases) to compensate for the lack of LIG3 is a particular characteristic of HEK 293 cells (or other virus-transformed, cancer-like cells) still awaits investigation. In this context, it is important to note that the expression of several genes involved in mtDNA maintenance (including *LIG3* and *TOP3A*) is lower in human primary fibroblasts compared with HEK 293 cells. Therefore, we hypothesize that the limited replication capacity of fibroblasts hinders efficient adaptation to increased mtDNA turnover in the absence of LIG3.

Second, HEK 293 cells might possess a more robust defense against oxidative stress than mouse embryonic fibroblasts. Baseline oxidative stress, as experienced by the cell under regular culturing conditions, could be sufficiently averted in HEK 293 cells but overcharge mouse embryonic fibroblasts. Our gene expression data, however, do not support this hypothesis. Nevertheless, the importance of anti-oxidative defense in general is underlined by the fact that we detected mitigated mtDNA damage in a *LIG3^−/−^* HEK 293 cell line (clone *b*) that showed transcriptional upregulation of genes involved in cellular redox homeostasis. We demonstrate that not only advanced intracellular scavenging of ROS but also the limited availability of extracellular oxygen results in reduction of mtDNA damage.

**Figure 6.**
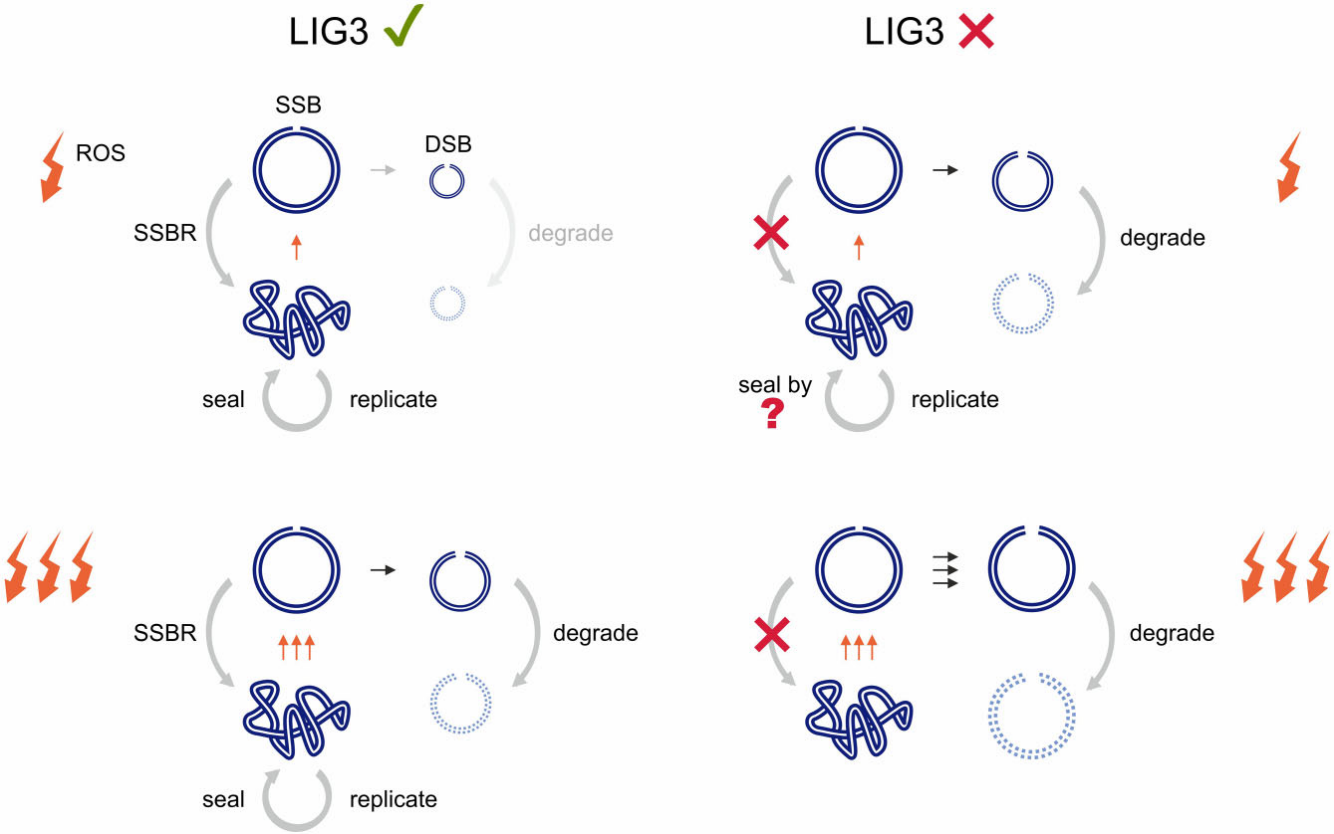
Proposed mechanism of mtDNA turnover in wild-type cells and in cells lacking LIG3 activity. Upper-left panel: Under regular conditions, mtDNA damage (arrows) is largely compensated by the LIG3-dependent repair of single-strand lesions (SSBR). Due to the low amount of DSBs, mtDNA degradation has a minimal impact. Lower-left panel: If extensive oxidative stress overloads the limited capacity of SSBR, SSBs are transformed to DSBs at higher frequency. Generated linear mtDNA molecules are not repaired but purged. Replication from intact mtDNA molecules can replace the missing copies. Upper-right panel: LIG3 deficiency leads to accelerated mtDNA degradation and reduced steady state levels of supercoiled mtDNA due to lack of SSBR. Compensatory replication is still sufficient to maintain required mtDNA copy numbers; however, the mechanisms that enables circularization of newly synthesized mtDNA molecules in the absence of LIG3 is unknown. Lower-right panel: In the absence of LIG3, extensive oxidative stress leads to abundant SSBs accompanied by massive DSB formation and mtDNA elimination. Since most copies of the remaining mtDNA carry SSBs or DSBs, replication is severely impeded, which altogether leads to mtDNA depletion.

In summary, we show here that loss of LIG3 prompts an accumulation of mtDNA SSBs at sites that are prone to subsequent formation of DSBs. The increased generation of DSBs leads to elevated mtDNA turnover due to accelerated degradation of linear mtDNA by a POLG/MGME1-dependent mechanism [2–4, 17] (Fig. 6). Specific SSBs and DSBs are detectable in *LIG3^−/−^* cells even without exposure to exogenous H_2_O_2_. We suggest that they originate from intrinsic oxidative damage since (i) an H_2_O_2_ pulse promotes the additional generation of breaks at the same sites, (ii) both SSBs and DSBs at these positions are attenuated in a *LIG3^−/−^* cell line that shows upregulation of anti-oxidative defense enzymes, and (iii) SSBs at these sites are reduced under conditions of low oxygen tension. It remains unclear whether SSBs at baseline or after oxidative challenge arise from direct backbone cleavage or BER-mediated base excision. In both cases, BER/SSBR enzymes are required, as direct cleavage also produces blocked 3′ termini needing processing before ligation [5].

While the role of oxidative damage in generation of mtDNA point mutations as one of the potential sources of long-term decline of mitochondrial function in aging has been challenged [37–39], our data implicate the relevance of oxidative stress for intrinsic mtDNA turnover, an often underestimated aspect of maintaining the integrity of the human mitochondrial genome.

## Supplementary Material

gkaf1000_Supplemental_File

## Data Availability

Sequence data generated in this study have been deposited in the Sequence Read Archive (SRA) with BioProject ID PRJNA1196334.
