# Supplementary material for: Ligase 3 prevents oxidative strand break-induced mitochondrial DNA loss but is not essential for replicative circularization
Source: Nucleic Acids Res. 2025 Oct 14;53(19):gkaf1000. doi: 10.1093/nar/gkaf1000 (PMC12526102; doi:10.1093/nar/gkaf1000)
Supplement: gkaf1000_Supplemental_File [file gkaf1000_supplemental_file.pdf]

Ligase 3 prevents oxidative strand break-induced mitochondrial DNA loss but is not essential for replicative circularization

Genevieve Trombly, Afaf Milad Said, Alexei P. Kudin, Kerstin Hallmann, Anano Kakabadze, Viktoriya Peeva, Kerstin Becker, Karl Köhrer, Gábor Zsurka and Wolfram S. Kunz

SUPPLEMENTARY INFORMATION

Supplementary Figures

Supplementary Figure S1: CRISPR-Cas9-mediated genetic inactivation of LIG3 in human HEK 293 cells.

Supplementary Figure S2: Time course of cytochrome *c* oxidase/citrate synthase activity ratio after a 1 mM H_2O_2 pulse.

Supplementary Figure S3: Identification of various mtDNA conformations by nickase treatment and Southern blotting

Supplementary Figure S4: Cell viability during the time course of hydrogen peroxide application in wild-type and *LIG3*$^{-/-}$ cell lines.

Supplementary Figure S5: Quantification of band intensities corresponding to different conformations of the mtDNA under baseline conditions in wild-type and *LIG3*$^{-/-}$ cells as detected by Southern blotting.

Supplementary Figure S6: Detailed view of mtDNA regions that show increased frequency of mtDNA single-strand breaks in *LIG3*$^{-/-}$ clone ***a*** cells.

Supplementary Figure S7: Expression of genes involved in oxidative defense and in mtDNA maintanence in various wild-type cells.

Supplementary Figure S8: Size distribution of DNA fragments detected by long-read deep sequencing in *LIG3*$^{-/-}$ clone ***a*** cells.

Supplementary Tables

Supplementary Table S1: Primers used in this study.

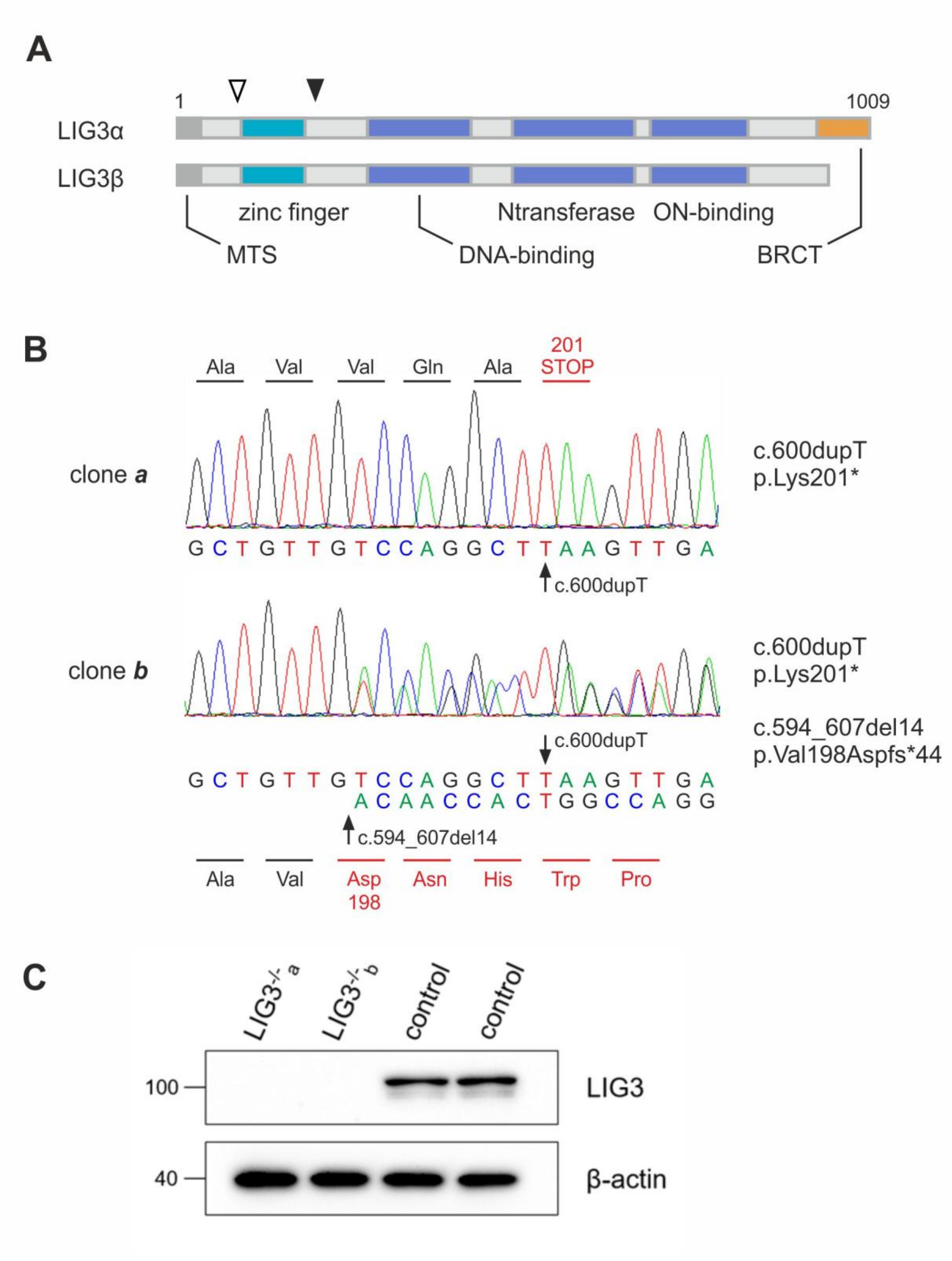

Supplementary Figure S1: CRISPR-Cas9-mediated genetic inactivation of LIG3 in human HEK 293 cells. (**A**) Schematic structures of LIG3 isoforms. The major isoform, LIG3α, is expressed in most tissues. LIG3β is an alternative splice variant, lacks the C-

terminal BRCT domain and is expressed in male germ cells. Due to an alternative start site (empty arrowhead) downstream of the N-terminal mitochondrial targeting sequence (MTS), a shortened, nuclear-targeted protein is also translated from both transcript isoforms, while the full-length protein is targeted to mitochondria. Black arrowhead, guide RNA targeting site. (**B**) Confirmation of truncating frameshift mutants by Sanger sequencing. (**C**) Western blotting demonstrating the lack of LIG3 protein in both $LIG3^{-/-}$ clones. β-actin serves as loading control. Molecular weight in kDa is indicated on the left side.

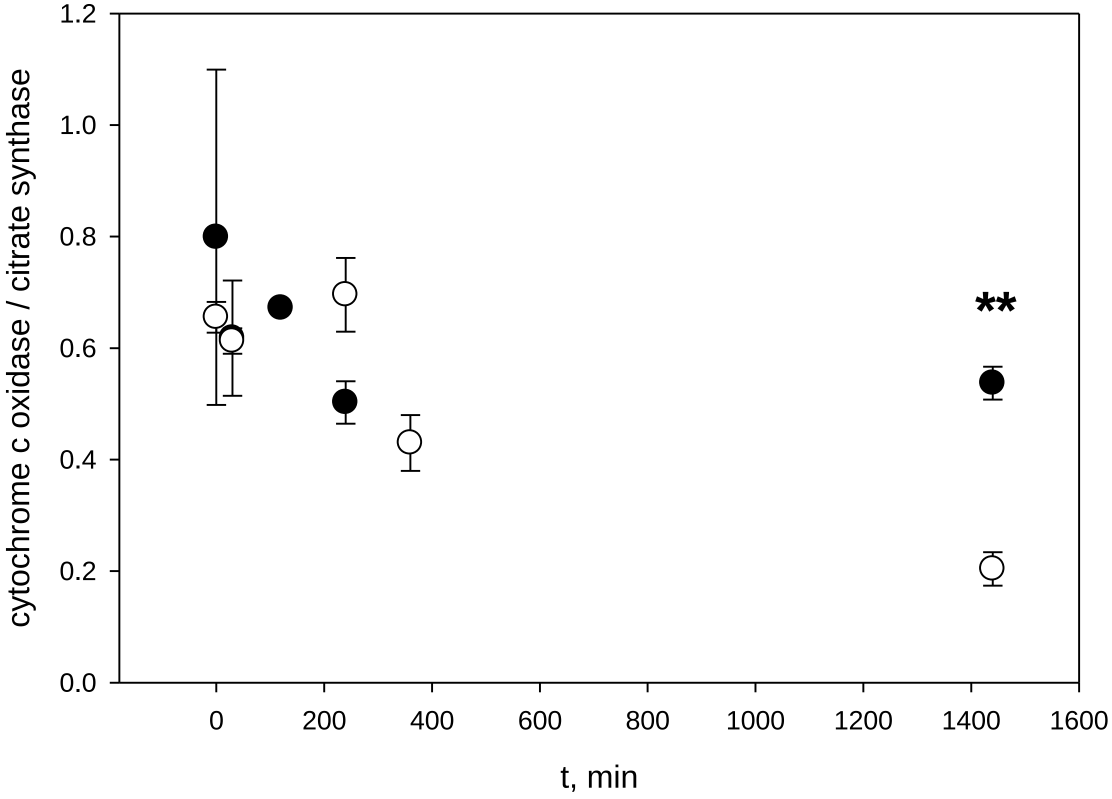

Supplementary Figure S2: Time course of cytochrome *c* oxidase/citrate synthase activity ratio after a 1 mM H_2O_2 pulse. The data are averages ± SEM of two independent experiments. Filled circles, wild-type cells; open circles, *LIG3*$^{-/-}$ clone ***a*** cells. **, $p < 0.01$, Student's t-test.

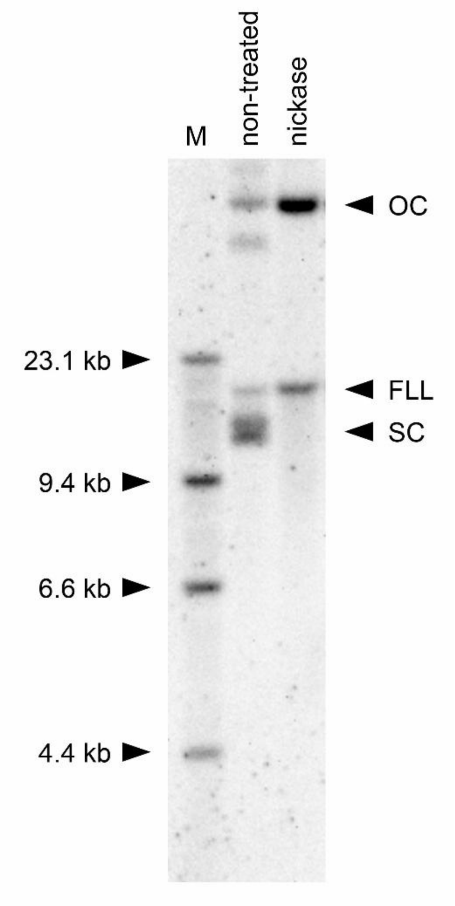

Supplementary Figure S3: Identification of various mtDNA conformations by nickase treatment and Southern blotting. Total cellular DNA was digested with Nb.BbvCI nickase that introduces a single-strand break after nucleotide position 13041. Note that fast-running supercoiled circular mtDNA (SC) disappears after nickase treatment, while open-circle (OC) becomes the most prominent conformation. The position of the band representing full-length linear mtDNA (FLL), corresponds to the size of the human mitochondrial genome (16.6 kb). M, molecular weight marker.

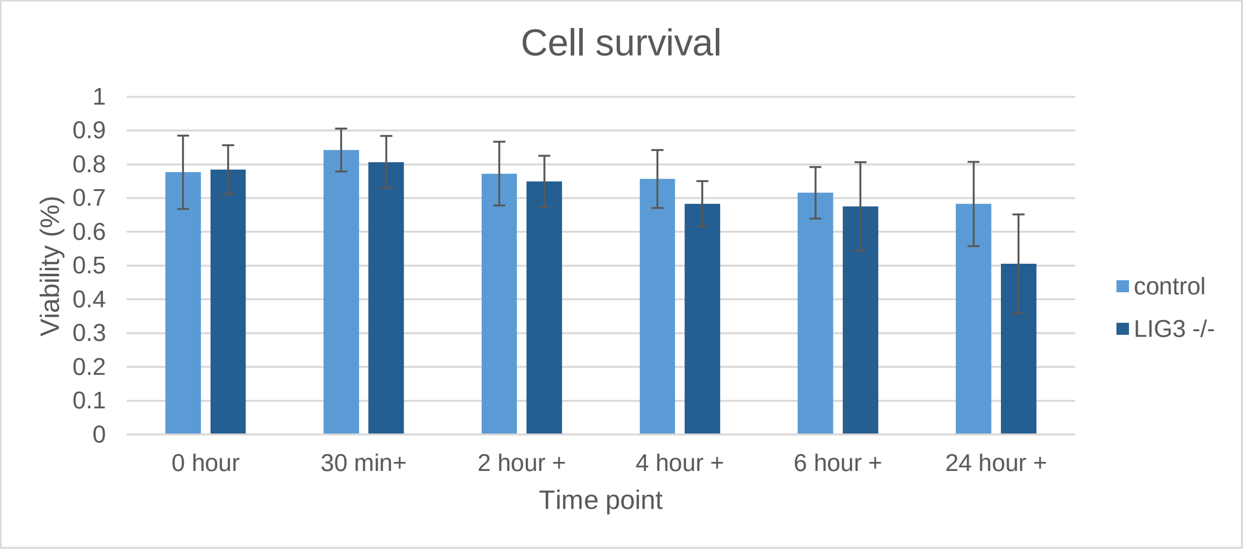

Supplementary Figure S4: Cell viability during the time course of hydrogen peroxide application in wild-type and *LIG3*$^{-/-}$ cell lines. Wild-type, n=15; *LIG3*$^{-/-}$ n=5. Error bars, standard deviation. The viability of cells remains constant throughout the experiment and there is no significant difference between the control HEK 293 cells and the knock-out cell lines.

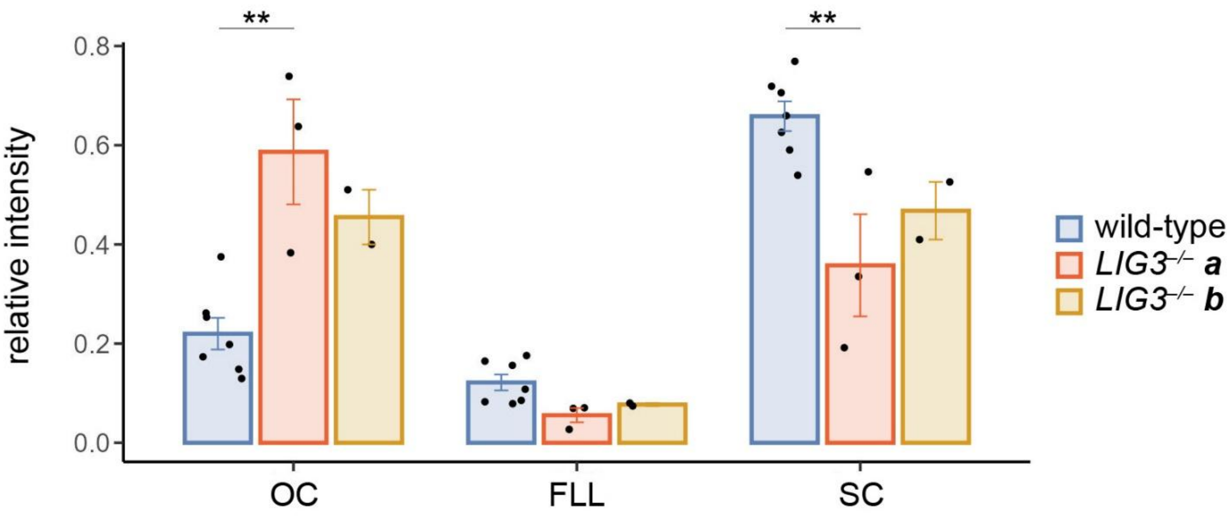

Supplementary Figure S5: Quantification of band intensities corresponding to different conformations of the mtDNA under baseline conditions in wild-type and *LIG3*$^{-/-}$ cells as detected by Southern blotting. OC, open circle; FLL; full-length linear; SC, supercoiled. Wild-type, n=7; *LIG3*$^{-/-}$ clone ***a***, n=3; *LIG3*$^{-/-}$ clone ***b***, n=2. Error bars, SEM. *, $p < 0.05$; **, $p < 0.01$; one-way ANOVA with Bonferroni post hoc test.

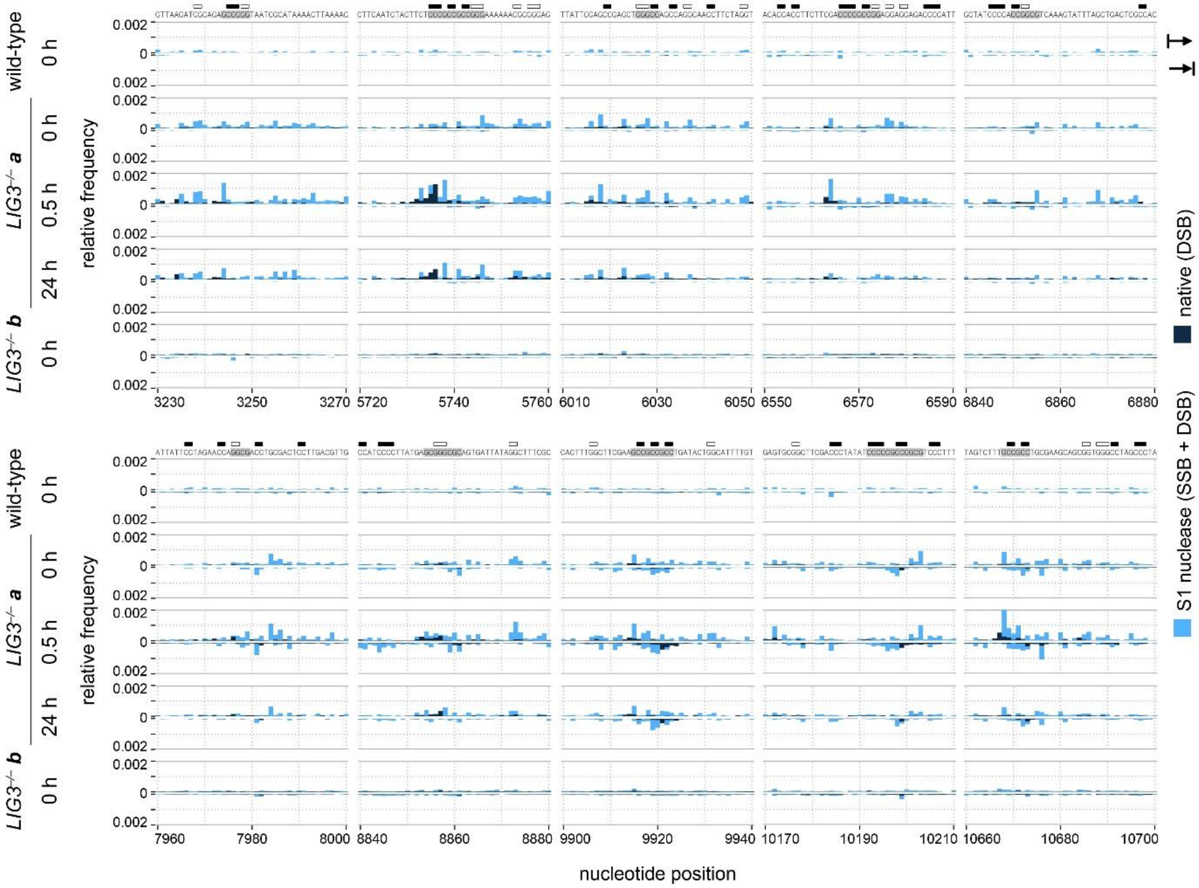

Supplementary Figure S6: Detailed view of mtDNA regions that show increased frequency of mtDNA single-strand breaks in *LIG3*$^{-/-}$ clone ***a*** cells. Ends were detected by long-read deep sequencing of DNA purified from enriched mitochondrial fractions. Upper panels show upstream ends (according to numbering of the human mitochondrial genome) and lower panels show downstream ends, as indicated on the top right side. Downstream ends are underrepresented in the first 5 regions due to vicinity to the linearization site and size exclusion during library preparation. Black, ends detected without S1 nuclease treatment (DSBs). Blue, ends detected in S1 nuclease-treated DNA (DSBs and SSBs). Corresponding sequences are shown on the top of the panels. Grey shading highlights GC-stretches. Filled and empty boxes above the sequences indicate sequence motives that can potentially be parts of G-quadruplex (G4) structures on the heavy and the light strand, respectively. 0h, sample before H_2O_2 treatment. 0.5 h, 24 h, time passed after the H_2O_2 exposure. Note the clustering of both SSBs and DSBs in *LIG3*$^{-/-}$ clone ***a*** cells even without H_2O_2 treatment.

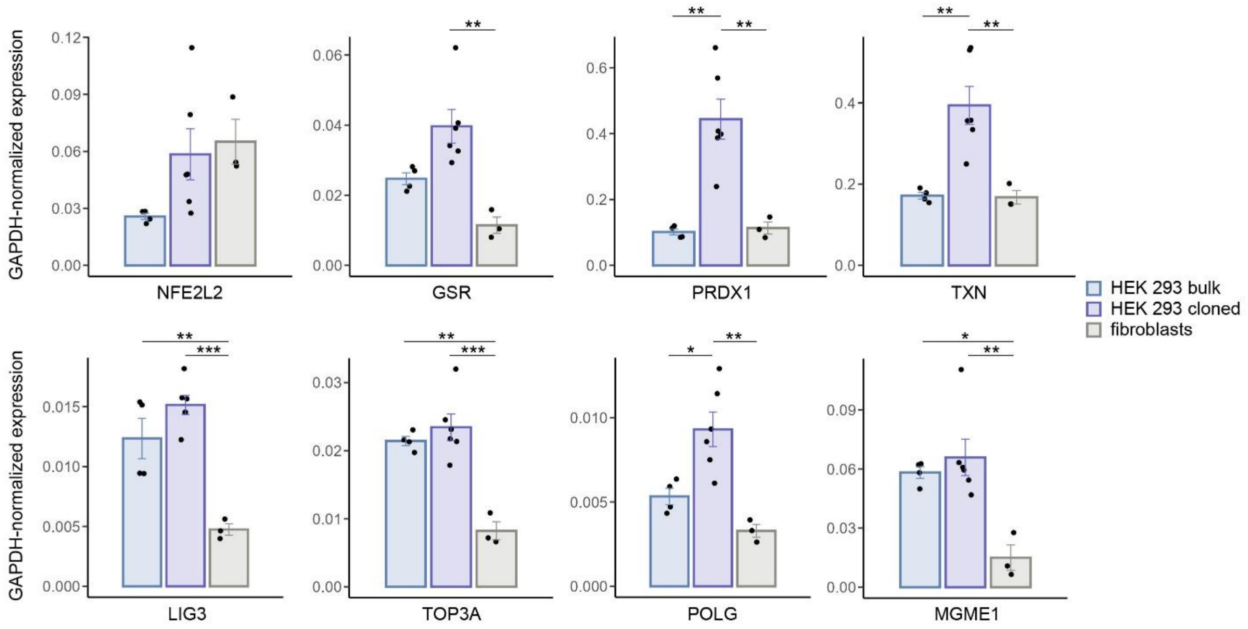

Supplementary Figure S7: Expression of genes involved in oxidative defense and in mtDNA maintenance in various wild-type cells. Transcript abundance was quantified by reverse transcription quantitative PCR and values were normalized to *GAPDH* expression. Error bars, SEM. *, $p < 0.05$; **, $p < 0.01$; ***, $p < 0.001$; one-way ANOVA with Bonferroni post hoc test. Note that a wild-type HEK 293 cell line that underwent single-cell cloning shows significantly increased expression of effectors of H_2O_2 detoxification (*PRDX1* and *TXN*). Human control fibroblasts (three different biological samples, cf. [22]) show lower expression of *TOP3A* and *MGME1* that are involved in mtDNA maintenance in comparison to HEK 293 cells. This points to a potential reduced replication reserve capacity in fibroblasts.

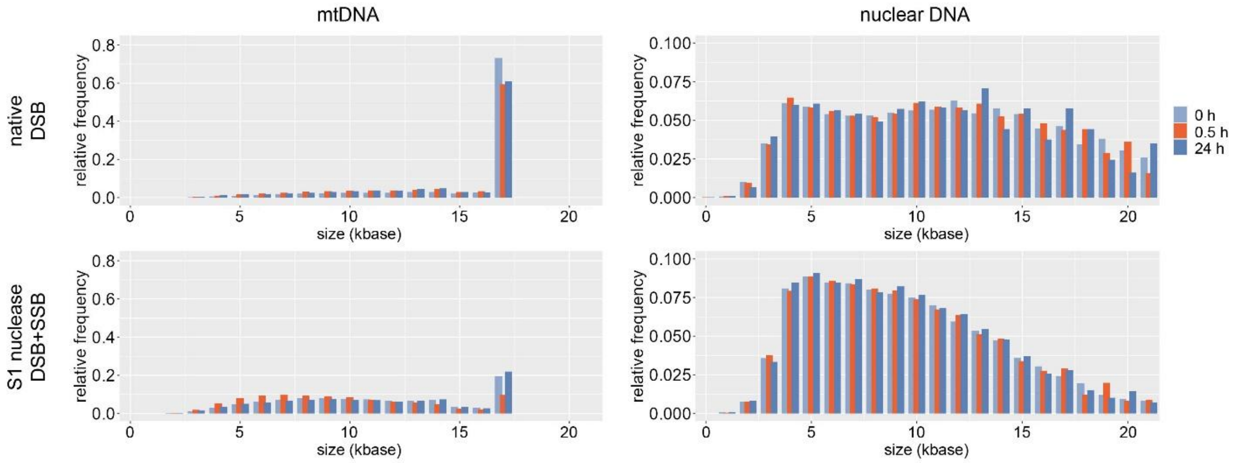

Supplementary Figure S8: Size distribution of DNA fragments detected by long-read deep sequencing in *LIG3*$^{-/-}$ clone ***a*** cells. DNA was isolated from enriched mitochondrial fractions and linearized by EagI restriction endonuclease. Left panels, mtDNA fragments; right panels nuclear DNA fragments. Top panels DNA samples without S1 nuclease treatment representing DSBs. Bottom panels, S1 nuclease-treated DNA additionally detecting SSBs. 0 h, before H_2O_2 treatment; 0.5 h, 24 h, time points after H_2O_2 exposure. In order to account for varying experimental size bias, mitochondrial and nuclear fragment counts were normalized to the nuclear fragment size distribution of an experiment with the highest proportion of large fragments. Fragments of size < 3 kb are underrepresented due to size exclusion during library preparation. mtDNA fragments can be detected 24 hours after H_2O_2 treatment, although, the majority of mtDNA is already eliminated at this time point. Note the increased frequency of short mtDNA fragments and the decreased frequency of full-length mtDNA 30 min after H_2O_2 treatment, which is not present in the nuclear DNA.

Supplementary Table S1

Primers used in this study

LIG3_gRNA_F	5’-CAGTGGTTGTCAACTTAGCCGTTTT-3’
LIG3_gRNA_R	5’-GGCTAAGTTGACAACCACTGCGGTG-3’
LIG3_g_F	5’-AGTGCTAGGGTAGTGACTTAACAAG-3’
LIG3_g_R	5’-AGCACAGAATTTTCACTCTTTAGCC-3’
LIG3_F	5’-CATGGTGAAGATCAGCAAGGAC-3’
LIG3_R	5’- GAGATCCCGTCAGCTGTATGAG-3’
MT-ND1_F	5’-GAACTAGTCTCAGGCTTCAACATCG-3’
MT-ND1_R	5’-CTAGGAAGATTGTAGTGGTGAGGGTG-3’
KCNJ10_F	5’-GCGCAAAAGCCTCCTCATT-3’
KCNJ10_R	5’-CCTTCCTTGGTTTGGTGGG-3’
NFE2L2_F	5’-TCCATTCCTGAGTTACAGTGTC-3’
NFE2L2_R	5’-GTGGACTACAGTTACCTACTTC-3’
PRDX1_F	5’-GACTTGTGTTGGGACTGCTGA-3’
PRDX1_R	5’-TGTAGTCAGACAGGCTGATATCT-3’
TXN_F	5’-ACGCTGCAGGTGATAAACTTGT-3’
TXN_R	5’-CTCTGAAGCAACATCCTGACAG-3’
GSR_F	5’-AGCTCTTCTTACTCCAGTTGCA-3’
GSR_R	5’-GAATGGCTTCATCTTCCGTGAG-3’
POLG_F	5’-ACCTCTTGACCAGGTGCATG-3’
POLG_R	5’-CATCCCAGTTGGGTTGGAAG-3’
MGME1_F	5’-TGGACTGTGTGGCTGAGTATC-3’
MGME1_R	5’-GAACCTGAAAGCTGTAGTTGGT-3’
TOP3A_F	5’-TGGACCTGAGGATTGGAGCT-3’
TOP3A_R	5’-GTAACTGATGAGCTGCTCTG-3’
GAPDH_F	5’-TCAGACACCATGGGGAAGGTGAA-3’
GAPDH_R	5’-GAATCATATTGGAACATGTAAACCATG-3’
MT-ND5_F	5’-TCATCCCTGTAGCATTGTTCG-3’
MT-ND5_R	5’-GAAGAACTGATTAATGTTTGGGTCT-3’
RNA18S_F	5’-GTTGGTGGAGCGATTTGTCT-3’
RNA18S_R	5’-GGCCTCACTAAACCATCCAA-3’

F, forward; R, reverse; underlined, overhangs introduced in gRNA primers
